# Measurement of economic resilience under the COVID-19 based on nighttime light remote sensing: Case of *Hubei* province

**DOI:** 10.1371/journal.pone.0307613

**Published:** 2024-09-27

**Authors:** Zhaoguo Wang, Xuechen Hao, Xishihui Du, Hua Ding, Zhiwei Xie

**Affiliations:** 1 College of Economic and Management, Shenyang Agricultural University, Shenyang, China; 2 519th of North China Geological Survey, Baoding, China; 3 School of Transportation and Geomatics Engineering, Shenyang Jianzhu University, Shenyang, China; Balochistan University of Information Technology Engineering and Management Sciences, PAKISTAN

## Abstract

This study investigates the economic resilience of cities in *Hubei* province during the COVID-19 pandemic, utilizing highway nighttime lights as a proxy indicator. By February 2020, the pandemic had caused a significant economic contraction in *Hubei*. However, by May 2021, a strong recovery was observed, with most cities experiencing growth rates of over 20%. Despite initially similar responses to the crisis, this study reveals significant heterogeneity in economic resilience across the examined cities in *Hubei*. The spatial distribution follows a core-periphery structure, with *Wuhan* exhibiting greater resistance to economic shocks compared to peripheral cities. Furthermore, the response capacity within the *Wuhan* urban agglomeration area exhibits regional variations. In summary, lockdown policies had spatially varied impacts on economic resilience across *Hubei*’s cities. These results offer valuable insights into regional economic resilience and contribute to the formulation of strategies aimed at effectively addressing future unforeseen events.

## Introduction

In December 2019, the COVID-19 broke out in *Wuhan*, China. Subsequently, the World Health Organization (WHO) declared COVID-19 as an international public health emergency [1, 2]. The virus quickly spread to various provinces within China and to other countries worldwide. Studies have provided evidence that the transmission of the coronavirus between individuals within family households and hospitals, as well as its spread across different cities, is indeed occurring [3, 4]. Government interventions, crucial for curbing the pandemic’s transmission, have presented a significant impediment to economic progress [5–7]. The COVID-19 epidemic had a noticeable adverse effect on GDP, resulting in an observable economic decline. Nonetheless, a recent study indicated substantial variation in the response to the pandemic among different cities [8].

Economic resilience denotes the inherent capability of an economy to effectively manage and overcome unforeseen shocks or disruptions, encompassing both resistance and post-event recovery [9, 10]. Cities with high economic resilience are more resistant to challenges from economic shocks [11, 12]. Evaluating the resilience of urban economies contributes to the comprehension of their capacity to withstand public health events and the conditions governing the development of policies in response to economic crises. Compared to previous economic crises, the ability of economic activities to respond promptly to the consequences of viral transmission and potential epidemics played a crucial role in determining economic resilience during the pandemic [13]. In the present context, examining the alterations in economic activities brought about by the COVID-19 pandemic can offer substantiation for regional economic resilience, thereby guiding the equilibrium between the impact of the pandemic and economic progress.

Measuring economic resilience is of utmost importance in obtaining a comprehensive understanding of the variations among regions in their capacities to effectively respond to the COVID-19 pandemic. Several frameworks and indicators have been developed to assess economic resilience [14–17]. Previous studies have employed diverse indicators to evaluate the economic resilience in the context of the COVID-19 pandemic [18, 19]. An alternative approach in assessing economic resilience involves the use of a core variable that directly measures cities’ response and recovery capacities in the presence of shocks. This methodology has gained significant popularity in the field. Relevant studies [20–23] primarily employ core variables, such as GDP, trade volume, employment, and fixed asset investment, to evaluate economic resilience from two aspects of economic resistance and recovery. Selecting core variables for economic resilience analysis during pandemics necessitates consideration of the specific economic system’s characteristics. Given the intrinsic link between transportation disruptions and economic stagnation during epidemics, the transportation sector emerges as a potentially valuable core variable.

The Chinese government responded swiftly to the COVID-19 pandemic with a lockdown policy and other economic measures that enabled the economy to be the first in the world to rebound from the epidemic. *Hubei* province, the pandemic’s epicenter in China, witnessed the most severe economic impacts but has since embarked on a noteworthy economic revival, warranting further investigation of its underlying recovery mechanisms. This study seeks to comprehensively assess the economic resilience of cities within *Hubei* province during the COVID-19 pandemic, with a specific focus on analyzing and visualizing fluctuations in the transportation sector as a key indicator of economic activity. To this end, nighttime light data and highway data were fused to describe highway traffic economics at the city scale. Taking into consideration the nature of economic resilience, the economic decline degree and recovery degree were quantified to assess economic resistance and recoverability. Finally, the economic resilience of cities was appraised by analyzing their associations with resistance and recoverability.

## Literature review

Traffic and transportation play a pivotal role in fostering economic development, with notable ramifications for sectors such as tourism, the built environment, the shared economy, and various other industries [24–26]. It is widely recognized that there exists a positive correlation between transportation and economic development [27]. Highways, which serve as the primary infrastructure for traffic and transportation, play a crucial role in facilitating population movement, attracting investments, and adjusting industrial structure [28–30]. By conducting an analysis of traffic patterns on highways, one can acquire significant insights into the economic conditions and the interconnectedness among cities. The COVID-19 pandemic has significantly influenced mobility, as evidenced by the implementation of road traffic restrictions in urban areas during the lockdown period. Analysis of remote sensing imagery and electronic surveillance data reveals a significant decline in traffic flow during the lockdown period. This observed reduction can be directly attributed to the imposed restrictions on the number of vehicles permitted on the road, highlighting the immediate impact of such measures on transportation activity [31, 32].

Nighttime remote sensing is a process using satellite sensors to observe urban building lights, ship lights, and burning biomass, providing supplementary information that is inaccessible through daytime remote sensing. This unique and intuitive perspective from space has found wide-ranging applications across various fields, including urban development, human activities, ecology and environment, as well as other pertinent social issues [33–36]. Nighttime lights (NTL) have been examined as a viable proxy indicator for economic activities, exhibiting a substantial association with crucial economic metrics, such as Gross Domestic Product (GDP) and Gross Regional Product (GRP) [37, 38]. Moreover, NTL data has the advantage of capturing intricate spatial patterns of economic activities compared to statistical data. Recent studies have also explored the use of NTL data for monitoring changes in urban economic activities during emergencies [31, 39, 40]. According to Roberts, Morocco’s overall light intensity exhibited a significant 10.9 percentage point monthly decline relative to its pre-crisis trend [41]. Additionally, NTL data offers a promising avenue for depicting transportation patterns as it effectively responds to vehicle lights [42, 43]. As a result, the utilization of NTL data can be crucial in the surveillance of economic fluctuations during the pandemic, thus providing a basis for evaluating the resilience of regional economies.

Despite the extensive research conducted on economic resilience, there is still a lack of consensus among scholars regarding its definition [9, 16, 44]. Economic resilience, an intrinsic characteristic of economic systems, revolves around their ability to endure and navigate disruptions. The ability to respond to a shock can be categorized into two main dimensions: resistance, which involves the capacity to minimize the impact of the shock, and recoverability, which pertains to the ability to recover from the shock. The capacity to react and adjust to economic shocks exhibits variation among different regions [8]. Variations in the scale of propagation, affected targets, and duration among different types of shocks can result in disparities in the attributes of economic resilience [45, 46]. The COVID-19 pandemic differs significantly from financial crises due to its nature as a life-threatening health shock. It is characterized by short-term multi-wave outbreaks [47, 48], which in turn lead to temporary and contextually contingent economic operations. Consequently, this study emphasizes the management of immediate economic challenges and ensuring the continuity of economic activities during and after the epidemic period. Accordingly, the concept of economic resilience in this study is defined as the capacity to maintain economic activities during the period of an epidemic and to recover and restore economic activities in the post-epidemic period. This definition encompasses both the ability to withstand economic shocks and the ability to bounce back and recover from them.

## Study area and data

### Study area

**Fig 1:**
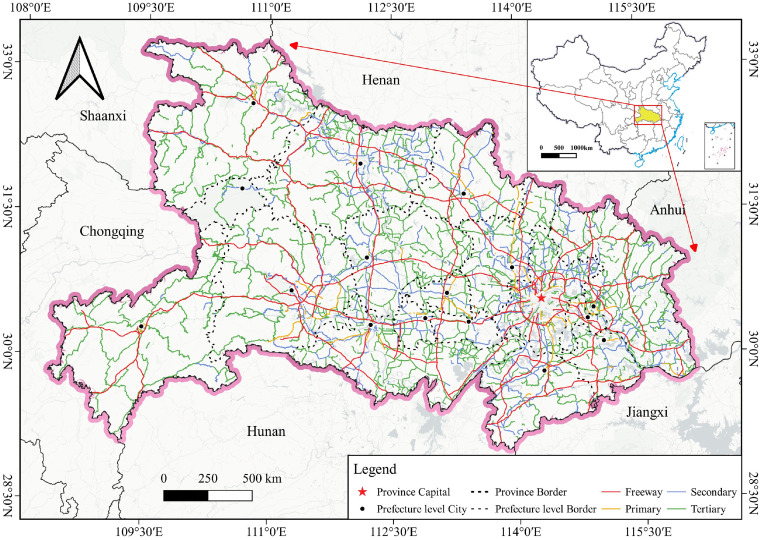
Overview of the study area. The map of China is derived from the standard map service system (http://bzdt.ch.mnr.gov.cn/), review number GS(2022)4314. The background map is acquired from the Basemaps. (https://basemaps.cartocdn.com/).

*Hubei* province, located within the middle reaches of the Yangtze River (108°21’42”E—116°07’50”E, 29°01’55”N—33°06’47”N) in central China (Fig 1), has a resident population of 58.30 million as reported by the 2021 Statistical Report of *Hubei* Provincial Bureau of Statistics, of which 37.36 million are in towns and 20.94 million are in villages. With Wuhan as its capital city, *Hubei* province comprises 17 prefecture cities, namely *Huanggang*, *Huangshi*, *Jingmen*, *Jingzhou*, *Qianjiang*, *Shengnongjia Forestry District*, *Shiyan*, *Suizhou*, *Tianmen*, *Wuhan*, *Xiantao*, *Xianning*, *Xiangyang*, *Xiaogan*, and *Yichang*. In the aftermath of the pandemic, *Hubei* province’s GDP in 2020 experienced a 5% decline compared to 2019. However, a rapid rebound was observed in 2021, with a 12.9% growth rate relative to 2020. At the end of 2021, the gross domestic product (GDP) of *Hubei* was 50012.94 billion yuan. Despite significant GDP fluctuations, *Hubei* witnessed continuous optimization within its industrial structure. The relative contributions of primary, secondary, and tertiary industries shifted from 8.4:41.2:50.4 in 2019 to 9.5:39.2:51.3 in 2020, further refining to 9.3:37.9:52.8 by 2021. Notably, the value-added output of the transportation industry experienced a 16.5% decline in 2020 compared to 2019. However, it exhibited a remarkable rebound in 2021, registering a 22.9% growth rate relative to 2020. *Hubei* province’s robust and well-positioned transportation system, recognized as “the thoroughfare of the nine provinces”, constitutes a vital link within China’s national network, and its transportation industry is among the best in the country. The highway length in *Hubei* is approximately 297,000 km, with 7,378 km of freeway. Moreover, the road transport industry in *Hubei* reported 74.475 billion person-km and 674.377 billion ton/km of goods transported in the same year. These metrics demonstrate the efficiency and importance of the provincial transportation system within the broader economic landscape.

### Data sources

#### Nighttime light data

This study utilizes monthly nighttime light (NTL) composites from February and May of 2019, 2020, and 2021. These composites were derived from the Visible Infrared Imaging Radiometer Suite’s (VIIRS) day/night band (DNB) by the Earth Observations Group (EOG). The EOG has made publicly available a suite of average radiance monthly composite images generated from VIIRS-DNB data through the Payne Institute for Public Policy at the Colorado School of Mines. The monthly composites of VIIRS provide near-global coverage with a spatial resolution of 15 arc seconds, corresponding to approximately 460 m^2^ at the equator [41]. This rich dataset, characterized by moderate spatial resolution and high temporal coverage, proves valuable for diverse earth observation applications. The version of “vcm” chosen for this study demonstrates superior data quality and eliminates any data that may have been affected by stray light.

#### Highways data

Highways are facilities with certain technical capacities for connecting cities, towns, villages, industrial and mining bases, etc., mainly for the use of automobiles. According to ‘The Chinese Technical Standard for Highway Engineering’ (JTG B01-2014), highways are divided into five levels: freeways, primary highways, secondary highways, tertiary highways, and township highways (Table 1). The research scope was restricted to the top four tiers of the national highway system, prioritizing major transportation arteries that underpin intercity movement and economic exchange [43]. Highway vector data at different grades were obtained from the National Catalog Service for Geographic Information (https://www.webmap.cn), 1:250000 National Basic Geographic Database (2017), and the geographic coordinate system is CGCS_2000.

**Table 1:** Highway design speed and width.

Highway levels	Design Speed (km/h)	Width (m)
**Freeway**	100-120	40-70
**Primary highway**	100-120	40-70
**Secondary highway**	60-80	30-60
**Tertiary highway**	40-80	20-40
**Township Highway**	Under 30	16-30

#### Auxiliary data

The provincial and prefecture geographic base maps of *Hubei* in the ArcGIS shapefile format were acquired from the Geographic Data Sharing Infrastructure, College of Urban and Environmental Science, Peking University (http://geodata.pku.eud.cn). The geographic coordinate system is CGCS_2000.

### Data processing

#### Nighttime light data

NPP/VIIRS nighttime light images were transformed to the Albers equal-area projection and clipped to encompass only the geographical extent of *Hubei* province. The effect of noise caused by cloud cover in the NTL data was mitigated by implementing a cloud mask to exclude cloudy pixels. Additionally, negative values were adjusted to 0, representing complete darkness. To create ‘the best’ highway NTL images, NTL data were intersected with highway data at various spatial resolutions.

#### Highways data

Four-tiered vector highway data (freeways, primary, secondary, and tertiary) were utilized in this study to determine the optimal pixel size for nighttime light image extraction. Highway density (HD) and highway pixel density (HPD) were combined to assess the appropriateness of the pixel size for each grade. HD was calculated as the ratio of the length of the highways to the area of the corresponding region, while HPD was calculated using Eq (2), utilizing the highway density of raster images. The HPD at different spatial resolutions was compared to the HD, and the optimal pixel size was determined based on the closeness of the two values.

$$\begin{array}{c} {\text{HD}}_{\text{j}}=\frac{L_{j}}{S} \end{array}$$ (1)

$$\begin{array}{c} {\text{HPD}}_{\text{j}}=\frac{n_{xj}}{N_{x}\cdot x_{j}} \end{array}$$ (2)

where {*HD*_*j*_, *HPD*_*j*_, *j* = 1,2,3,4} are the HD and HPD of the *j*-grade highway, *j* = 1 denotes the freeway, *j* = 2 denotes the primary highway, *j* = 3 denotes the secondary highway, *j* = 4 denotes the tertiary highway, *L*_*j*_ denotes the total kilometers of the *j*-grade highway, *S* denotes the total area of the study area, *x*_*j*_ denotes the resolution of the *j*-grade highway, *n*_*x*_*j* denotes the pixel number of the *j*-grade highway at a certain resolution *x*, and *N*_*x*_ denotes the total pixel number of the corresponding nighttime light image at a certain resolution *x*.

**Fig 2:**
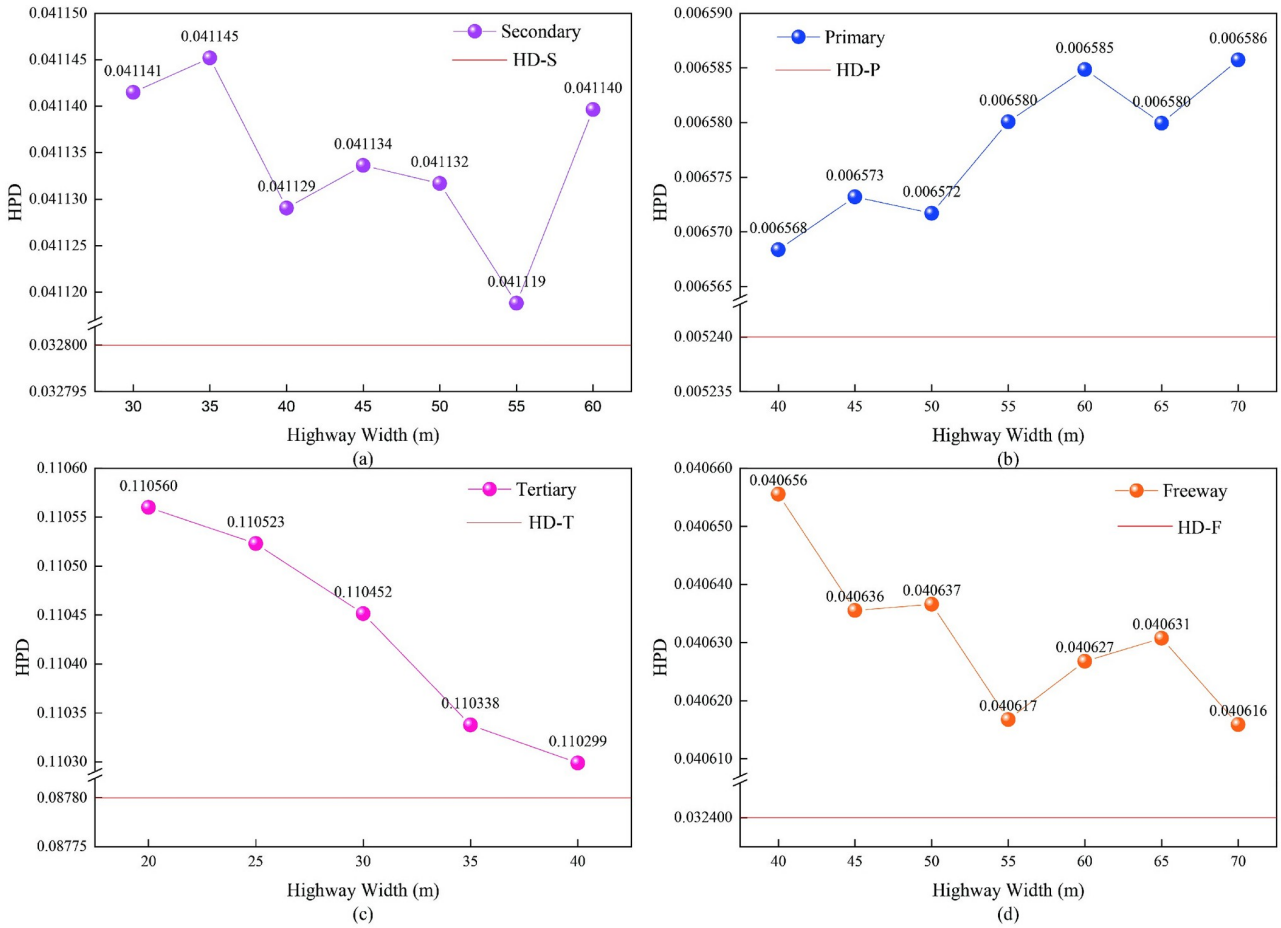
The HPD for the four highway grades with different pixel sizes: (a) freeways, (b) primary highways, (c) secondary highways, (d) tertiary highways.

**Fig 3:**
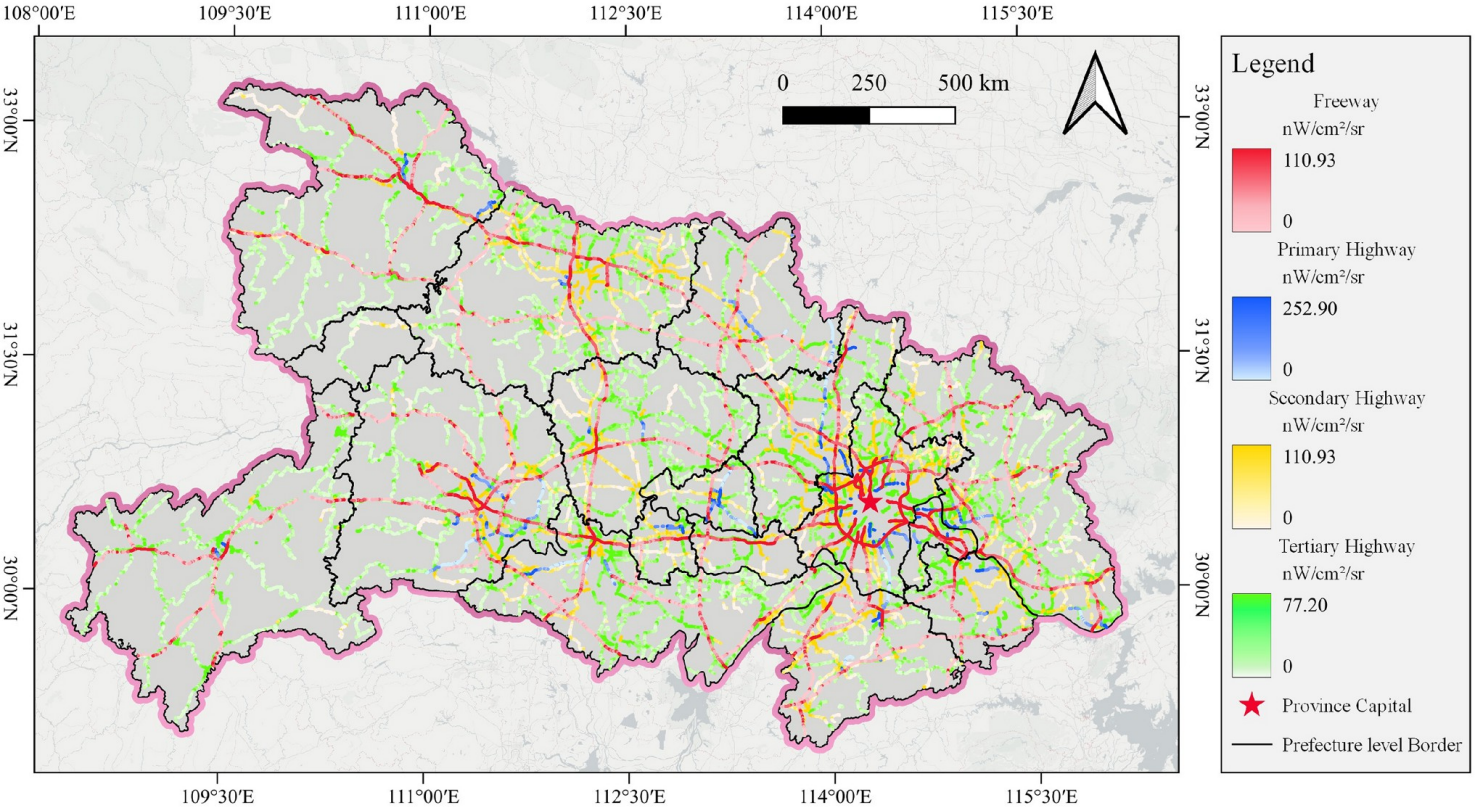
The highway nighttime light map in February 2020. The background basemap is from the Basemaps. https://basemaps.cartocdn.com/.

The data for each of the four highway grades were converted into raster images with different resolutions based on the width in Table 1. Then, the HPD with different resolutions for the four highway grades was calculated, as shown in Fig 2. The optimal pixel sizes for the four highway grades were 70 m (freeways), 40 m (primary highways), 55 m (secondary highways), and 40 m (tertiary highways). Therefore, highway nighttime light maps were produced by intersecting the NTL data with the optimal pixel size for the four highway grades. The highway nighttime light map in February 2020 is shown in Fig 3.

## Methods

This methodology is articulated in three stages. In stage 1, NTL and highway data were integrated to generate highway nighttime light maps and to calculate highway nighttime traffic prosperity indices (HNTPIs). Subsequently, in stage 2, the economic decline index and recovery index were proposed to quantify the resistance and recoverability of economic resilience. Finally, in stage 3, the economic resilience of cities during the COVID-19 pandemic was evaluated and ranked in terms of resistance and recoverability using a four-quadrant diagram and biplot.

### Highway nighttime traffic prosperity index

The highway nighttime traffic prosperity index (HNTPI) was proposed to quantitatively evaluate highway traffic prosperity with NTL data using a highway-oriented method that can directly describe the traffic flow at night and has a strong correspondence with socioeconomic parameters [43]. In this study, the HNTPI was used to quantitatively describe highway traffic activities at the city scale and reveal the dynamics of the traffic economy in *Hubei* during the COVID-19 pandemic. A city with a larger HNTPI means a more prosperous transport economy, and vice versa. HNTPI was obtained from total nighttime lights (TNL) for different highway grades from the highway nighttime light map:

$$\begin{array}{c} \text{HNTPI}=\frac{1}{4}\sum_{j=1}^{4}\frac{\sum_{i=0}^{n-1}\frac{DN_{ij}}{DN_{jmax}}}{n_{j}} \end{array}$$ (3)

where {*DN*_*i*_*j*, *i* = 0,1,…,*n*-1, *j* = 1,2,3,4} is the *i*th DN value of the *j*-grade highway, *j* = 1 denotes freeway, *j* = 2 denotes primary highway, *j* = 3 denotes secondary highway, *j* = 4 denotes tertiary highway, and *n*_*j*_ denotes the pixel number of the j-grade highway from highway nighttime light images.

### Economic decline and recovery index

During the COVID-19 pandemic, economic resilience focused specifically on fluctuations in short-term economic operations. Due to the implementation of various control measures and economic policies, economic characteristics were diverse in the different stages of the COVID-19 pandemic. To obtain the whole picture of economic fluctuations, the pandemic was divided into an epidemic period (2020) and a post-epidemic period (2021). Because all cities in *Hubei* province experienced a policy change from lockdown to the restoration of normal movement during the epidemic, the lockdown stage (February 2020) and the unblocking stage (May 2020) were selected for study in this period. Moreover, the same months in 2021 were used as paired matches for the recovery stage in the post-epidemic period.

Following the methodology of Martin et al. [21], the HNTPI was utilized as a direct indicator to quantitatively assess economic resilience. Without the impact of the epidemic, the HNTPI from the same month in 2019 was selected as the benchmark from which to calculate the economic decline index and recovery index. The degree of economic decline is represented by the decline in the HNTPI in 2020 as a percentage of 2019 levels, while the degree of economic recovery was expressed by the recovery in HNTPI in 2021 as a percentage of the same month in 2019. A higher economic decline index indicates that the economy was more affected by the epidemic, while a higher economic recovery index indicates that the economy recovered favorably. The formulas for the indices are calculated by the following:

$$\begin{array}{c} {\text{Decline}}_{\text{m}}=\frac{HNTPI_{m}^{t+1}-HNTPI_{m}^{t}}{HNTPI_{m}^{t}}\times 100\% \end{array}$$ (4)

$$\begin{array}{c} {\text{Recovery}}_{\text{m}}=\frac{HNTPI_{m}^{t+2}-HNTPI_{m}^{t}}{HNTPI_{m}^{t}}\times 100\% \end{array}$$ (5)

where {*Decline*_*m*_, *Recovery*_*m*_, *t* = 2019}, are the economic decline and recovery index for city *m* for a given year; $HNTPI_{m}^{t}$, $HNTPI_{m}^{t+1}$, and $HNTPI_{m}^{t+2}$ represent the HNTPI for city *m* in February/May of 2019, 2020, and 2021, respectively.

To express economic resistance and recoverability within a given timeframe, we have defined these terms as the average performance during economic decline and recovery, respectively. Notably, resistance was chosen to be negatively correlated with the economic decline index. The greater the resistance/recoverability is, the more resilient/recoverable the economy is likely. The formulas for resistance and recoverability are as follows:

$$\begin{array}{c} {\text{Resistance}}_{\text{m}}=-\frac{\sum {Decline}_{m}}{2} \end{array}$$ (6)

$$\begin{array}{c} {\text{Recoverability}}_{\text{m}}=\frac{\sum Recovery_{m}}{2} \end{array}$$ (7)

### GGE biplot

Biplot methodology is a statistical technique introduced by Gabriel [49], and it provides a graphical analysis for multivariate data. GGE Biplot, an extension of classical biplots, is a comprehensive analysis system for graphically interpreting the interaction between the target variable and explanatory variables [50, 51]. Hence, a GGE biplot was used to graphically rank cities by their economic resilience in *Hubei* during the COVID-19 pandemic by simultaneously analyzing their resistance and recoverability.

## Results

### Economic fluctuations during COVID-19

#### Degree of economic decline

**Fig 4:**
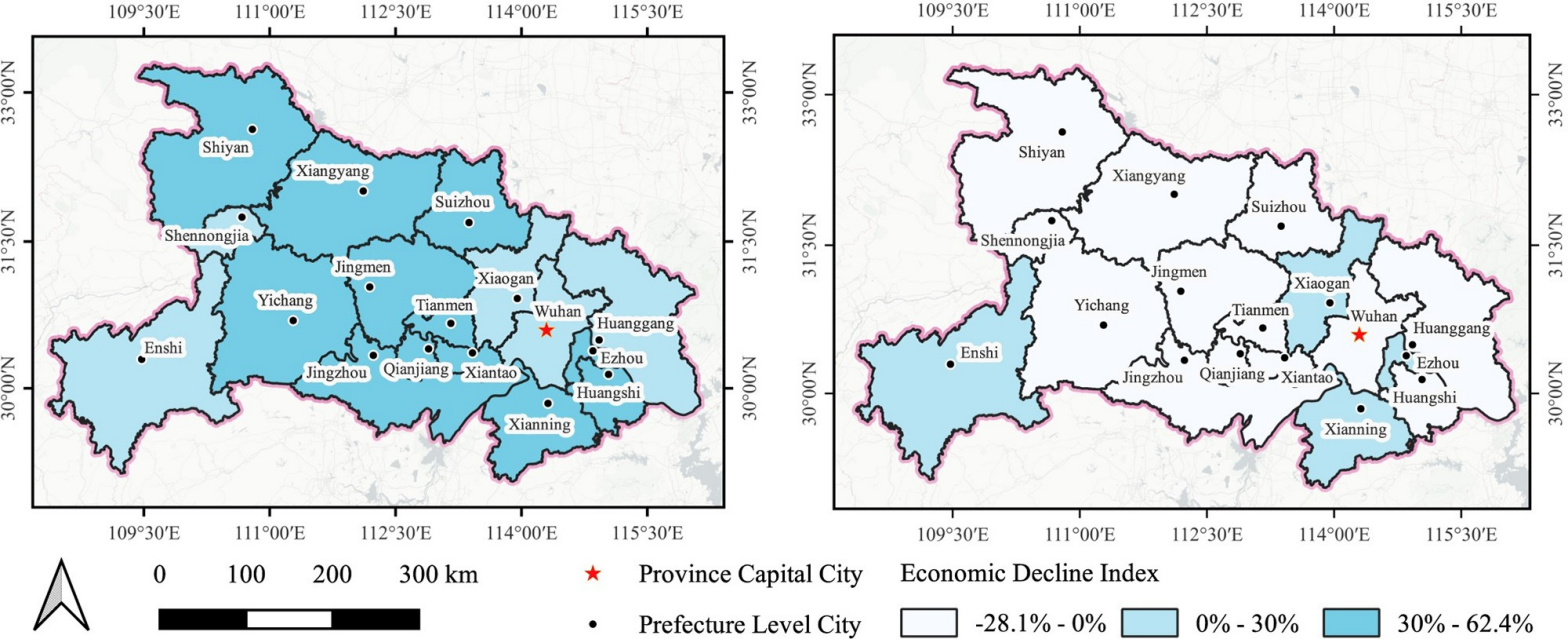
Economic decline degree of cities in February 2020 (a) and May 2020 (b). The provincial and prefecture geographic base maps were acquired from the Geographic Data Sharing Infrastructure, College of Urban and Environmental Science, Peking University. https://opendata.pku.edu.cn/.

Fig 4 offers evidence pertaining to the extent of economic downturn experienced during both the period of lockdown and the subsequent easing of restrictions in response to the epidemic. It is evident that the ongoing pandemic has significantly influenced the progress of regional economic development. During the lockdown phase in February 2020, the economy encountered an abrupt and significant decline, characterized by evident regional disparities. Fig 4a indicates that the central region bore the brunt of the economic decline, whereas the peripheral areas were relatively less affected. Notably, a significant decline exceeding 30% was observed in twelve cities situated in the central region. Among these cities, *Xiantao* exhibited the most substantial decrease, with a decline of 62.36%. In contrast, cities situated in the eastern and western regions of *Hubei*, such as *Enshi* and *Shennongjia*, experienced relatively smaller decreases in various ranges from 0% to 30%. This phenomenon can be ascribed to the topographical characteristics of the region, characterized by its mountainous terrain and dense forest cover, coupled with the isolated nature of these locations. Additionally, minimal decreases were noted in the eastern cities of *Wuhan*, *Huanggang*, and *Xiaogan*. The most surprising outcome was observed in *Wuhan*, the epicenter of the initial COVID-19 outbreaks, where the economic decline was the smallest at 3.51%. Despite the varying degrees of economic loss experienced by these cities, all of them displayed considerable economic contraction. In February 2020, the economy was in a stagnant phase, and the contraction in these cities was consistent with anticipated trends.

**Fig 5:**
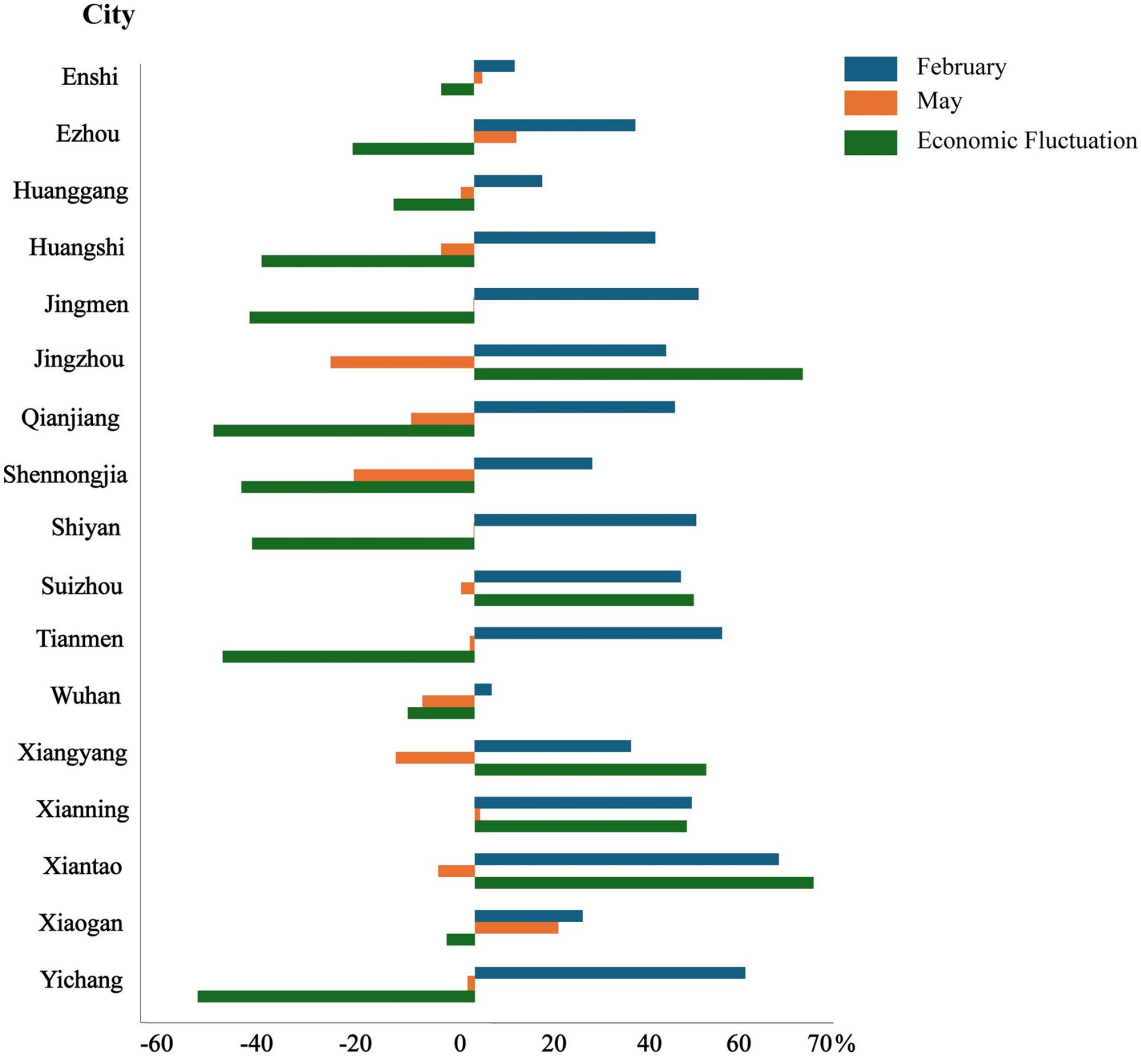
Economic decline indices of each city in the epidemic period.

By May 2020, it became apparent during the unblocking phase the economic downturn in *Hubei* province had been effectively alleviated as a result of the gradual relaxation of lockdown measures. Notably, economic growth was observed in thirteen cities, as depicted in Fig 4b. These cities were mainly situated in the central and eastern regions of *Hubei* province, exhibiting an economic decline index below 0%. Among them, *Jingzhou* reported the most notable growth rate, experiencing a substantial increase of 28.08%. Despite the lifting of the city closure, four neighboring cities to *Wuhan* still encountered a slight economic downturn. Fig 5 reveals a substantial decrease in the extent of economic downturn across most urban areas, with declines approaching 50% in several cases. In fact, the data suggests a substantial rebound in the economy amidst the period of decline. From February to May 2020, cities such as *Xiaogan*, *Enshi* and *Wuhan* exhibited a relatively low volatility, with fluctuations remaining under 15%. The results highlight a noteworthy polarization of economic dynamics between the lockdown and unblocking stages, particularly in cities located within the central region of *Hubei* province. During the lockdown phase, these cities underwent substantial economic contractions, which were subsequently followed by noteworthy expansions in economic activity as the process of unblocking commenced. Despite these upswings, overall recovery fell short of pre-pandemic levels, suggesting residual economic effects of pandemic control measures.

#### Degree of economic recovery

**Fig 6:**
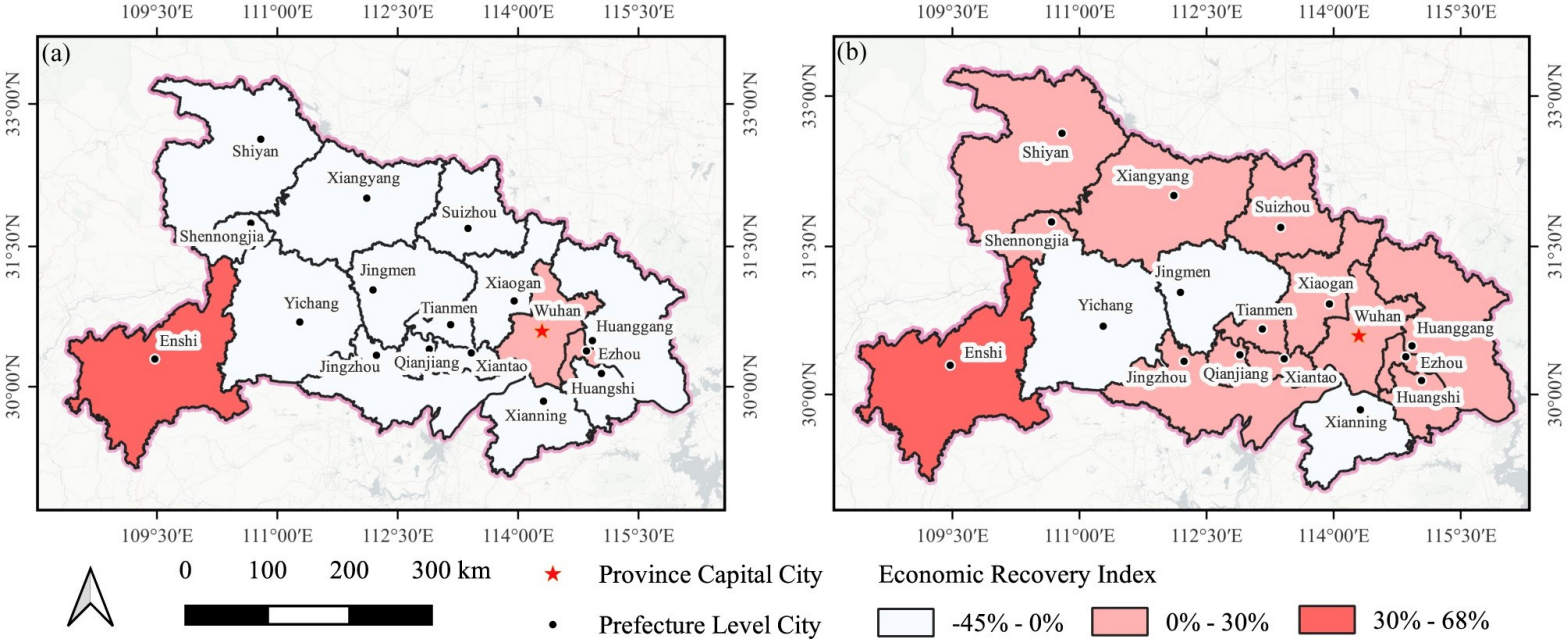
Economic recovery degree of cities in February 2021 (a) and May 2021 (b). The provincial and prefecture geographic base maps of *Hubei* were acquired from the Geographic Data Sharing Infrastructure, College of Urban and Environmental Science, Peking University. https://opendata.pku.edu.cn/.

Fig 6 provides a visual representation of the spatial heterogeneity in economic recovery across the study area, enabling analysis of the geographic patterns and disparities in post-pandemic economic performance. Fig 6a reveals a concerningly sluggish economic recovery within the central region of *Hubei* province, as indicated by an economic recovery index falling below 0. In February 2020, *Jingmen* encountered a significant economic downturn, marked by a substantial decline of 45.02%. This decline serves as a reflection of the dire economic circumstances prevailing in the *Hubei* province during that critical period. Moreover, a contrasting pattern of notable economic expansion was observed in *Enshi*, *Wuhan*, and *Ezhou* in February 2021 compared to the corresponding period in 2019. *Enshi*’s economy experienced the most significant surge, exhibiting a growth rate of 67.86%. Similarly, *Wuhan* and *Ezhou* exhibited growth rates of 16.57% and 12.31%, respectively. The positive economic trajectory can be ascribed to the close geographic proximity and well-developed transportation networks, which have facilitated the recovery process.

**Fig 7:**
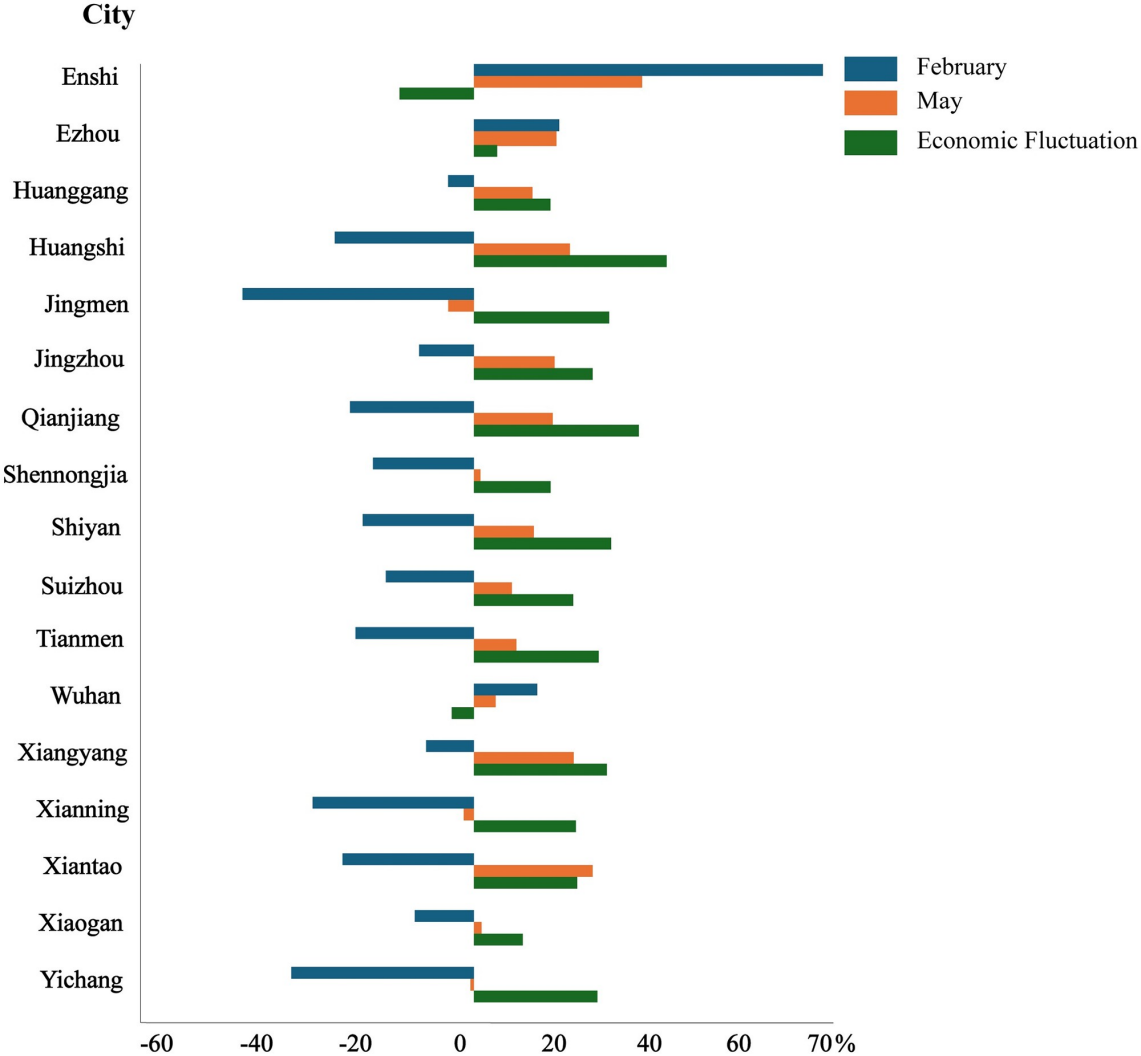
Economic recovery indices of each city in the post-epidemic period.

By May 2021, a significant improvement in economic recovery was evident, as the majority of cities witnessed a surge of more than 20%, as shown in Figs 6b and 7. Emerging from the economic recession, most cities in *Hubei* province demonstrated a trajectory towards comprehensive expansion. Notably, *Enshi* exhibited exceptional growth, exceeding all other cities with a remarkable range of 2% to 28%. This led to a remarkable overall increase of 47.02% compared to two years earlier. It is worth mentioning that *Huangshi* had the highest rate of economic fluctuation, amounting to of 53.9%, with *Qianjiang* following closely behind. Despite efforts to stimulate economic growth, the cities of *Jingmen*, *Yichang* and *Xianning* continued to face economic recession, with *Jingmen* being the most severely affected in terms of its recovery. Despite the challenges faced, there is a positive perspective to consider. The economic recovery index in May 2021 showed a significant improvement, nearing the levels observed in 2019. Despite temporary dips in *Enshi* (-20.84%) and *Wuhan* (-6.24%), *Hubei* province exhibits a promising trend of gradual and consistent economic recovery across its urban areas, highlighting its overall promising trajectory towards sustained economic growth.

#### The pattern of economic fluctuations

**Fig 8:**
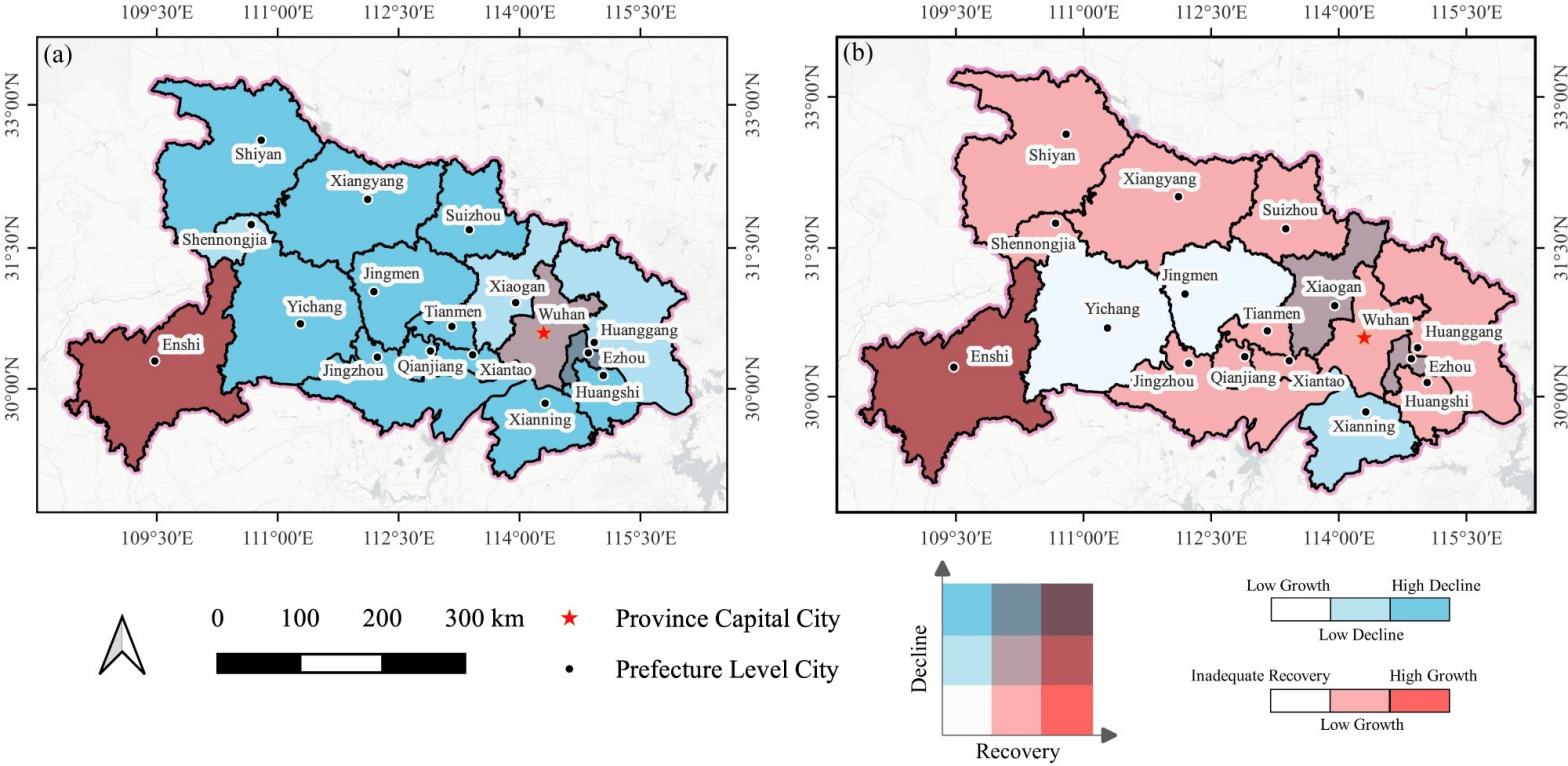
Bivariate choropleths for the economic decline index and recovery index in February (a) and May (b). The provincial and prefecture geographic base maps of *Hubei* were acquired from the Geographic Data Sharing Infrastructure, College of Urban and Environmental Science, Peking University. https://opendata.pku.edu.cn/.

To comprehend the dynamics of urban economic fluctuations in the period pre- and post-epidemic periods, the economic decline index and the recovery index were employed and combined for further investigation. The aforementioned indices were stratified into three classifications, as depicted in Figs 4 and 6. These classifications were then synthesized to generate a 3x3 bivariate choropleth map, as presented in Fig 8. Each index was assigned a distinct color saturation, which varies in a gradient manner to correspond with increasing values. The color gradient transitions from lighter to darker hues to indicate different levels of economic performance. Specifically, with respect to the economic decline index, these gradations signify low growth, low decline, and high decline. Similarly, in the context of the economic recovery index, the gradations range from inadequate recovery to low growth to high growth.

A bivariate choropleth map (Fig 8) offers a comprehensive depiction of the combined distribution of economic decline and recovery. In Fig 8a, the province is predominantly characterized by regions colored in blue, indicating that despite the significant economic downturn observed in February 2020, the majority of cities had not yet regained their pre-pandemic levels by February 2021. This suggests a prolonged adverse effect of COVID-19 on the economy. *Enshi* and *Wuhan* emerge as notable regions characterized by their vibrant and strong economic expansion, as evidenced by their red-dyed status. These areas experienced only marginal economic downturn in February 2020. Fig 8b, conversely, is characterized by predominantly red-dyed regions, which signify extensive economic growth and a stage of full recuperation in the majority of cities. This observation indicates a favorable and motivating pattern in the overall economic recovery of the *Hubei* province. Notably, *Enshi* has demonstrated a consistent trend of transitioning from a period of low decline to one of high growth, thereby emphasizing its exceptional economic resilience. Conversely, *Xianning*, *Yichang*, and *Jingmen* displayed a relatively sluggish rate of recovery, despite demonstrating indications of economic improvement by May 2020. The bivariate choropleth map (Fig 6) provides a significant visual depiction of the diverse degrees of economic impact resulting from the COVID-19 pandemic.

### Economic resilience of cities in *Hubei*

**Fig 9:**
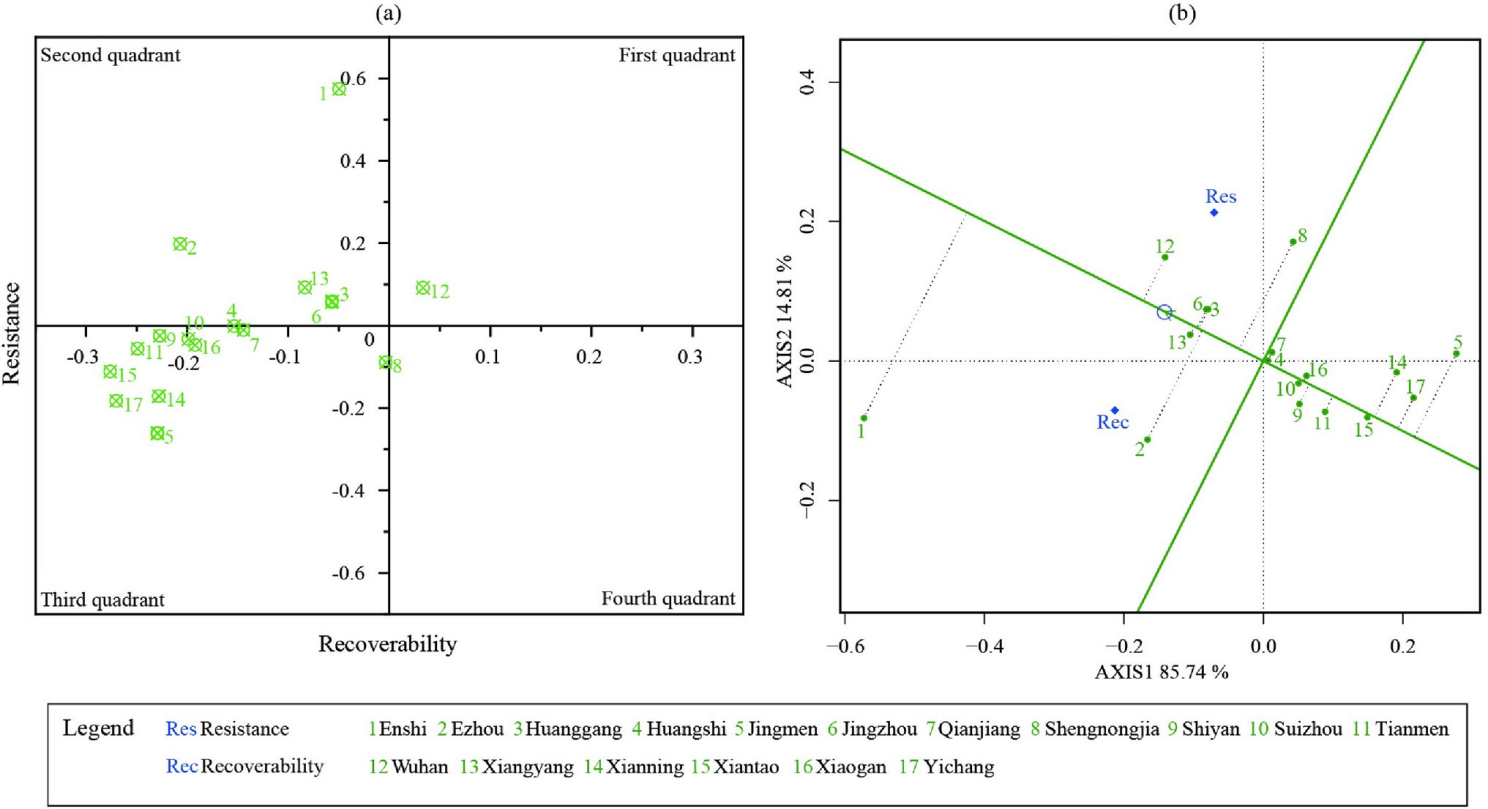
The four-quadrant diagram of economic resistance and recoverability (a) and biplot of economic resilience (b).

Building upon the theoretical framework of economic resilience, this study devised a novel four-quadrant diagram and biplot based on the key dimensions of resistance and recoverability (Fig 9). Fig 9a reveals that the majority of *Hubei*’s cities clustered within the second and third quadrants, indicating a shared level of initial shock resistance but potentially greater economic instability in the face of sustained crises. *Wuhan* stands as a noteworthy exception to this pattern, occupying the first quadrant of the framework. This positioning signifies a demonstrably superior level of economic resilience compared to other cities, characterized by both robust resistance to initial shocks and a swiftness in economic recovery. Within the second quadrant, the cities such as *Enshi*, *Ezhou*, *Huanggang*, *Jingzhou*, and *Xiangyang* exhibited a notable pattern of low resistance but high recoverability. However, closer examination revealed significant heterogeneity among these cities in terms of their economic recoverability. Despite the inherent challenges associated with mitigating the impact of unforeseen crises, these cities can still achieve swift recovery by implementing efficient measures. *Enshi* emerged as a standout case, exhibiting a demonstrably higher level of economic recoverability compared to its counterparts within this quadrant. Conversely, cities located in the third quadrant exhibited a concentrated arrangement within the range of -0.28 to -0.15 for resistance, and from -0.20 to 0 for recoverability. This implies that the ability to withstand economic shocks poses a significant challenge for these cities, and the subsequent recovery process is equally arduous. Notably, *Shennongjia* emerged as the sole region exhibiting a notable capacity to endure economic adversity.

Furthermore, GGE biplot in R studio [52] was utilized to graphically rank the economic resilience of cities in the second and third quadrants, taking into account their overall performance in terms of resistance and recoverability during the COVID-19 pandemic (Fig 9b). The biplot graph was measured by the average environment coordinate (AEC), with the AEC abscissa line indicating higher resilience and the origin representing average resilience. As depicted in Fig 9b, five cities located in the second quadrant exhibited resilience levels that surpassed the average. *Enshi*, *Xiangyang*, *Jingzhou*, *Huanggang*, and *Ezhou* exhibited varying degrees of economic resilience in descending order. Despite exhibiting a relatively strong capacity for economic recovery, *Ezhou* demonstrated a deficiency in economic recoverability. Notably, there was a gap in economic resilience among cities in the third quadrant, despite their similar performances in terms of economic resistance and recoverability. *Shennongjia* stood out as the only city to surpass the average resilience. The remaining cities were ranked in terms of economic resilience as follows: *Qianjiang* and *Huangshi* were at the same level, followed by *Suizhou*, *Xiaogan*, *Shiyan*, *Tianmen*, *Xiantao*, *Xianning*, *Yichang*, and *Jingmen*.

## Discussion

In the assessment of economic resilience, it is of utmost importance to select a proper variable that captures the characteristics of the economic system and its interconnections with other regions [16]. *Hubei* province is notable for its transportation system, which effectively links various geographically separated regions. The COVID-19 lockdown measures in *Hubei* province significantly impacted mobility and transportation systems, leading to a strong correlation between transportation disruption and subsequent economic stagnation [53, 54]. Therefore, assessing economic resilience during an epidemic by focusing on the transportation sector is a valid approach. To capture the immediate and dynamic fluctuations in the transportation economy that may not be accurately reflected in government statistics, nighttime light data was utilized due to its high spatial and temporal resolution [43]. Accordingly, this study employs nighttime light data to evaluate the economic resilience of cities in *Hubei* province during the COVID-19 pandemic, with a specific focus on the impact of transportation-related economic changes.

The COVID-19 pandemic exposed a heterogeneous regional response in terms of resilience, characterized by a core-periphery structure. *Wuhan*, exhibiting a high urbanization rate, large population, strong economic base and infrastructure, and exceptional medical resources, demonstrated remarkable economic resilience, particularly in its resistance to the initial shock [55]. However, *Wuhan* was susceptible to a rapid spread of the virus and frequent outbreaks of the epidemic due to its dense road network and economic connections, which hindered its recovery [56]. Small cities with a simple economic system tend to recover more quickly from shocks [57], as evidenced by the sharper rebound of *Enshi* than that of others during different periods, contributing to its remarkable recoverability.

Furthermore, metropolitan areas were more resilient than intermediate [58], while there was diversity in economic resilience within urban agglomerations. In the *Wuhan* urban agglomeration (WUA), there was a noticeable spatial pattern of economic resilience, in which the east displayed higher resilience than the western region. *Huanggang* and *Ezhou* exhibited substantial economic resilience, featuring advantageous recoverability. *Wuhan* has a superior ability to radiate to other cities, with a distinct spatial interaction with *Ezhou*, *Huanggang* and *Huangshi* [59], which form a critical area defined by a one-hour commuter circle. These cities, particularly *Huanggang* and *Ezhou*, experience economic benefits resulting from the phenomenon of agglomeration around *Wuhan* [60, 61]. This agglomeration contributes to the overall economic strength of the eastern region, surpassing that of the western region, and plays a significant role in driving their economic resilience. In contrast, other cities exhibited a pattern of low resistance and low recoverability. *Xianning*, *Xiantao*, and *Tianmen*, with lower economic resilience, occupied relatively low positions and poor interactions with the WUA, thereby experiencing less of an economic spillover effect [59, 62, 63]. Generally, urban agglomerations, by facilitating internal connectivity and fostering a diverse economic specialization, can significantly bolster regional economic resilience and promote sustainable growth [64]. Robust internal linkages and a well-integrated economic structure allow regions to recover more effectively from diverse shocks.

Moreover, spatial heterogeneity in economic fluctuations during the COVID-19 pandemic highlights economic disparities. Drawing on economic resilience theory, this study introduces a novel four-quadrant diagram and biplot based on resistance and recoverability, facilitating a nuanced analysis of these variations. These disparities can be attributed to variations in the economic base, infrastructure, local policy interventions, and other related factors [21, 65, 66]. Diversified economic performance was observed at various stages, particularly in February and May 2020, underscoring the influence of control measures and local policies on economic activities. This observation aligns with the viewpoint expressed by Ezcurra and Rios [67]. Building on prior research [68, 69], this study confirms that industrial structure significantly influences economic resilience, shaping a region’s ability to absorb and recover from shocks. Notably, a positive correlation exists between industrial diversification and urban economic resilience [70]. Cities with a more diversified industrial base are better equipped to withstand disruptions and demonstrate stronger resilience, as exemplified by *Wuhan*’s case.

This study introduces a novel perspective for exploring the economic repercussions of the outbreak and evaluating urban economic resilience. While it sheds light on this phenomenon through the lens of nighttime light data, further research would be significantly enhanced by the utilization of longer-term NTL data series. Furthermore, the integration of traditional statistical data would contribute to a more comprehensive understanding of the complex economic impacts of the pandemic. In upcoming research, the utilization of NTL data to analyze variations in economic resilience across different regions and scales could provide deeper insights into the economic effects of the COVID-19 pandemic in China. Moreover, by incorporating an analysis of COVID-19 policies, it would be possible to evaluate the effectiveness of different measures, thereby offering valuable support for future decision-making in emergency management.

## Conclusion and policy implication

The case study of *Hubei* province, which served as the epicenter of the pandemic, offers a particularly illustrative example and valuable insights into the formulation of strategies to enhance economic resilience in cities in the future. In this study, we explored the spatial characteristics of the economic resilience of cities in *Hubei* province amidst the COVID-19 pandemic by using highway and nighttime light data. This innovative approach adds depth to the analysis of economic resilience within the framework of transportation advantages and the immediate consequences of of the pandemic.

Evaluating regional economic resilience provides valuable insights into a region’s strengths and weaknesses in responding to unforeseen events. This study offers key findings: (1) Highway nighttime lights effectively captured the spatial heterogeneity of economic fluctuations throughout the pandemic. (2) The pandemic exposed a core-periphery structure in economic resilience, with a strong central core and weaker outlying areas. (3) *Hubei* province exhibits significant variation in city-level economic resilience, highlighting the need for tailored policy responses to address diverse vulnerabilities. (4) Resilience within the *Wuhan* urban agglomeration is higher in eastern regions compared to western regions. Despite challenges, *Wuhan* displayed remarkable economic resilience. (5) Lockdown policies, reflected by evolving transportation accessibility, had spatially varied impacts on economic resilience across *Hubei*’s cities.

Strengthening regional economic resilience during periods of shocks and disruptions necessitates the development of targeted strategies. Policymakers should prioritize balanced regional development by mitigating disparities between core and non-core cities. Core cities should focus on facilitating spillover effects and fostering resource and information exchange with other cities to stimulate their growth, thereby establishing a unified economic entity. Non-core cities, on the other hand, should focus on enhancing industrial resilience, ultimately reducing disparities with core cities and promoting cohesive regional development. Additionally, diversifying the economic structure and fostering collaboration among industries are crucial for balanced regional development. Furthermore, improving transportation networks, particularly in the western region, is essential to ensure efficient regional connectivity and foster economic network connectivity. This enhanced road infrastructure will optimize resource allocation. Finally, establishing long-term monitoring and evaluation mechanisms is necessary to gain valuable insights into the dynamic fluctuations in regional economic resilience over time. This will provide policymakers and stakeholders with a better understanding of the effectiveness of strategies and interventions aimed at promoting long-term economic sustainability.
